# "Card sorting": a tool for research in ethics on treatment decision-making at the end of life in Alzheimer patients with a life threatening complication

**DOI:** 10.1186/1472-684X-10-4

**Published:** 2011-03-03

**Authors:** Lionel Pazart, Chrystelle Vidal, Didier Faivre Chalon, Sophie Gauthier, Florent Schepens, Elodie Cretin, Jean-Louis Beal, Pierre Pfitzenmeyer, Régis Aubry

**Affiliations:** 1 Clinical Investigation Centre, Inserm CIT 808, Besançon University Hospital, 2, Place St Jacques, 25030 Besançon, France; 2 Pain & Palliative Care Department, Besançon University Hospital, Boulevard Fleming, 25030 Besançon, France; 3 Burgundy Geriatric network, Champmaillot Geriatrics Centre, 2, rue Jules Violle, 21079 Dijon, France; 4 Georges Chevrier Center, UMR CNRS 5605, University of Burgundy, 4, Boulevard Gabriel, 21000 Dijon, France; 5 Palliative Care Centre, La Mirandière, 1, rue Gouge, 21800 Quetigny, France; 6 Geriatrics Department, Champmaillot Geriatrics Centre, 2, rue Jules Violle, 21079 Dijon, France; 7 Faculty of Philosophy, 'Logiques de l'Agir', EA2274, University of Franche-Comté, 30, rue Megevand, 25030 Besançon, France

## Abstract

**Background:**

End stage dementia is a particularly difficult aspect of care for patients with Alzheimer's disease and related dementias. In care institutions, caregivers and family are concerned by treatment decision-making for an acute life threatening complication occurring in Alzheimer patients at the end of life. How should the best treatment pathway be decided: to treat or not to treat? Which arguments are used for decision-making? These are mainly ethical questions which are currently difficult to express and investigate.

**Methods/Design:**

Cross sectional multicentre study of clinical cases involving 67 health centres (university hospitals, general hospitals, local hospitals and homes for the elderly) in the east of France. The method was based on the "card sorting" technique, with a set of 36 cards, each labelled with a different item relating to arguments for treatment decision-making. For each clinical case, medical staff and carers expressed in a meeting the pieces of information which they believed had been taken into account in the decision. Each participant received a card game, selected fewer than ten and ranked them according to the importance they attached to each one. All selected cards were then put on the table anonymously for participants, respecting the order of importance of the cards in each pile. Lastly, all games were photographed together in order to analyse occurrence and order frequencies. The cards were then classified on the table by frequency to open the discussion. Discussion time, which was conducted by the head carer of the department, concerned the clinical situation of the patient based on the shared responses.

**Discussion:**

During team meetings, the "card sorting" method was quickly adopted by professionals as a tool to assist with discussion beyond the context of the study. The participants were not compelled to mention their feelings in relation to a case, and it is significant that the anonymity which we tried to maintain so that each person felt "listened to" without value judgement was very often discarded by the individuals themselves.

## Background

End stage dementia is a particularly difficult aspect of care for patients with Alzheimer's disease and related dementias.

Faced with an acute life threatening complication in a patient suffering from advanced Alzheimer's disease who cannot express his/her will in an informed manner, doctors, care teams and families are confronted with the dilemma of which is the least bad decision in relation to the person's interests. Should an assessment be undertaken? Should a treatment be initiated? Should ongoing treatment be changed? Should the patient with dementia be transferred to another hospital? Which arguments could be used to support the decision concerning introducing or withholding, withdrawing or continuing treatment for an acute complication occurring in people at the end of life when ethical issues are paramount?

This situation leads to carers and relatives calling into question the relevance, efficiency and above all the use of treating this complication. At the crossroad of medical, psychological, philosophical and moral reflection, this involves complex and fundamental questioning on respect for life and for the person [1,2]. How can a happy medium be found between abandon and excessive therapeutic intervention? [3,4] How can a decision be reached which respects the person and which may require us to administer additional treatment, or to continue or withdraw treatment already in place? [5] When confronted with a compulsory choice, all doctors, care teams and families then face the double difficulty of responsibility and doubt.

Concerning the determinants of decision-making, besides applying the main ethical principles [6], several publications [1,2,4,5,7-10] focus on taking directives into account, the role of the person of trust, the need to coordinate teamwork and the participation of carers in the decision-making process, and some others show the need to take into account the religious and moral beliefs of people as well as the cost of care and treatments [11,12].

Benett & al. recently highlighted the lack of emphasis on research which informs clinical decisions in end of life care [13]. In a recent review of the different methods used by researchers in the end of life domain [14], the predominant use of qualitative or mixed methods which called on social science tools was highlighted (interview, focus group, Arts/drama, Quality of life tools/surveys, Storytelling, Narratives/diaries, Mixed methods).

The role of caregivers (healthcare professionals and family) in the decision-making process must be understood before designing a research protocol on this topic. Their role depends on socio-cultural aspects, organisational aspects, professional guidelines and legislation.

In France, if a patient is in an advanced or terminal phase of a severe and incurable disease, or if a patient receives only artificial life sustaining treatment, French law (Code of Public Health, Law No. 2005-370 of 22 April 2005 on patients' rights and end of life) allows the clinician caring for the patient to limit or stop unnecessary or disproportionate treatment. The decision is purely the responsibility of the clinician in charge of the patient, but it must be made after discussion with the care team and with a medical consultant outside the department. The patient's physician must seek and take into account any previous directives made by the patient, and obtain the opinion of the family or relatives. So far in practice in France, dialogue within teams seems poorly organised and reported, and often limited to discussions among physicians, as in oncology. Advanced directives are not yet sufficiently widespread in France, especially on the situation of people with Alzheimer's disease or related illnesses.

The participative aspect of the decision-making process is therefore often lacking in such complex clinical cases, and must be adapted to the hierarchical relationships within the team, which tends to limit the ability of nursing care professionals to express themselves in front of the clinician.

To address this essential matter in research, the analysis of ongoing clinical situations seems to us the most pragmatic approach. Sharing the different practical experiences of numerous teams concerning dementia patients at the end of life may help to establish markers for strengthening the decision to introduce or withhold, withdraw or continue treatment for an acute complication.

## Methods/design

This is a cross sectional multicentre study of clinical cases concerning all medical and medico-social institutions admitting people with advanced dementia, in an area with a population of 2.2 million in the east of France (Burgundy and Franche-Comté).

Of the 92 institutions contacted, 67 (72.8%) responded favourably to our request (University hospitals, general hospitals, local hospitals and homes for the elderly).

The protocol was approved by the clinical ethics committee of Besançon University Hospital.

This study was funded by the National Clinical Research Programme (PHRC) of the French Ministry of Health.

Each department was invited to consider all eligible patients suffering from advanced dementia of the Alzheimer type presumed to be at the end of life (presenting with cachexia and more rapid change in their general state over the last three months) and presenting with acute complications which may endanger life and challenge the relevance of continuing, changing or withdrawing, introducing or not introducing a treatment likely to alter survival: organic or systemic infection resisting a first line treatment; occurrence of probable pulmonary embolism; pending stroke; phase IV obliterating arteriopathy of the lower limbs usually requiring deobstruction, a bypass or an amputation; heart failure occurring in treated congestive heart failure; acute kidney failure; respiratory decompensation occurring in treated respiratory failure; signs of appearance or progression of cancer.

The study concerned the patients present in the departments and those who had died in the two months prior to the study.

A clinical research assistant initially met the reference person from the health institution for the study in order to find out, with the help of the medical file, the aspects of the questionnaire relating to the pathological situation of the patients studied, their environment and current therapeutic situation (withholding, introducing, withdrawing or continuing treatment) for the intercurrent complication(s).

Data gathering on information taken into account by carers in the argument for their therapeutic decision was based on the patient file and by questioning carers in groups using the "card sorting" method.

Medical staff and carers involved in the treatment discussion and/or the treatment decision were compelled to express which information they believed had been taken into account in the decision for each clinical case. This was done with the help of a game involving 36 cards. Each card represents a piece of information which they believed had been taken into account in the decision.

One of the critical stages of the card sorting method consists in establishing the list of relevant headings to appear on the cards. We based an initial list of headings on information from the literature and a brainstorming session in the palliative care team of the principal investigator (led by a person who was independent of the department).

**Figure 1:**
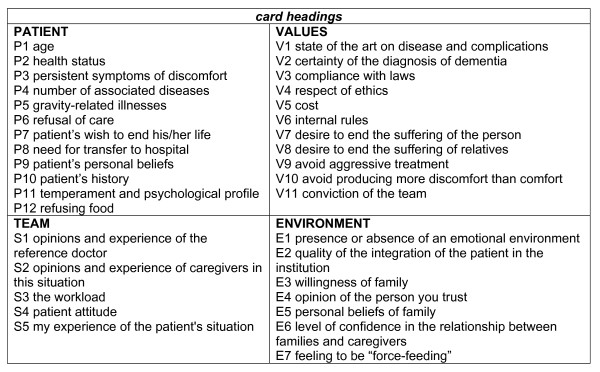
**Set of cards, each labelled with an item which could be an argument in decision-making**.

Based on this initial work, a pilot feasibility study was conducted in three different departments for 6 patients. At the end of this pilot study, we were able to consolidate the procedure since all participants had understood the "rules of the game" and adhered to the method, and four large families of decisive factors and 36 titles (see figure 1) were retained. Two "jokers" (blank cards) completed the game to replace information not necessarily initially foreseen in the game.

For each patient included in the study, collecting arguments took place during a meeting of medical and care staff involved in the decision to withdraw or continue, introduce or withhold treatment. The time necessary for studying a patient's situation was compatible with the availability of participants (20 minutes on average). Each situation was examined during a meeting around a table, in 3 phases:

➢ Recall time, led by the study reference carer, of the clinical situation in which the question was raised as to whether or not to introduce, continue, withdraw or withhold a treatment. The therapeutic decision (either implicit or explicit) was recalled.

➢ Then each participant received a "card game", with each card representing a piece of information which could be an argument in decision-making. Each participant selected the information (maximum of ten cards) which he/she believed had been taken into account in the decision and ranked the cards according to the importance which he/she attached to it (with the most important on the top of the pile). The clinical research assistant collected each pile from the participants, and put it in such a way that the function of each participant could be identified (using the sundial positioning strategy in order to recognise the position of each participant around the table).

**Figure 2:**
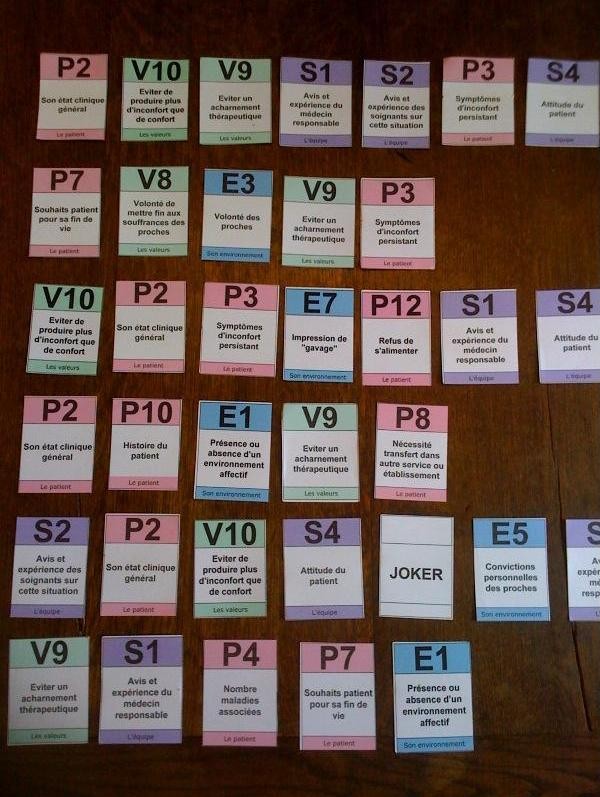
Example of selected cards put on the table, respecting the order of importance of each.

➢ All selected cards were then put on the table anonymously for participants, respecting the order of importance of the cards in each pile (see Figure 2). Lastly, all games were photographed together. This photograph later helped with data entry for the statistical analysis.

Depending on the wishes of the team, the cards were then classified on the table by frequency to open the discussion. Another option was to select only the first four ranked cards from each participant's pile. Discussion time, which was conducted by the head carer of the department, concerned the clinical situation of the patient based on the shared responses.

A descriptive analysis of all questionnaires was carried out (amount of available data, average, median, standard deviation, minimum, maximum or percentage, depending on the type of variable). The variables of the investigation were analysed using adapted statistical tests according to the nature of the variables (univaried: Variance analysis or Kruskall-Wallis test for the quantitative variables and chi-square test for qualitative variables) in order to determine those which were associated with the "decision" (5 modalities: Withdrawal of ongoing treatment, continuation of ongoing treatment, change in ongoing treatment, introduction of a treatment, non-introduction of a treatment).

Decision variable modalities were then merged into two modalities for the therapeutic situation of the complication(s) ("treated" group and "non-treated" group) for a more powerful analysis. The merge was established as follows:

- Treated group = continuation or modification of treatment during the complication(s), or introduction of a treatment.

- Non-treated group = withdrawal of ongoing treatment or non-introduction of a treatment.

The significance threshold of the statistical tests was fixed at 5% and formulation of the hypotheses was two-sided. The statistical analysis was carried out using SAS software for Windows Version 9.3 (SAS Institute, Inc., Cary, NC).

The statistical analysis of the "card games" used classical statistical tests:

- a univariate analysis allowed cards to be classified by frequency of appearance, then according to their level of importance using a scale from 0 to 10 (0 = non-cited, 10 = cited in first position, 9 = cited in second position, etc.).

- a bivariate analysis allowed the partial correlations between cards to be explored.

- a multivariate analysis, such as a multiple correspondence analysis, investigated the representation of the relationship between the decision on whether or not to introduce, continue, withdraw or withhold a treatment and the cards. This analysis was done by using the PROC CORRESP procedure of SAS software version 9.1.3 (SAS Institute, Inc., Cary, NC).

The analysis of the discussion after the cards were put on the table has not yet been carried out.

The results of the card game analysis must be examined in relation to the results of the characteristics of the patient's situation. This comparison will help to interpret the results of the card games (based on subjective declarations) according to the objective and factual elements from the patient's file.

## Discussion

Card sorting is a method of organising contents which is often used in the realm of the internet [15]. The use of card games is a relatively old and well accepted concept in medicine for testing patients' capacities, particularly in psychology, and in psychiatry [16] and used in medical training as a pedagogical tool [17,18]. More recently, card sorting appeared in the domain of palliative care as a tool for facilitating communication with the patient to approach end of life conditions [3]. Card sorting allows the way in which users rank and group together contents which are presented to them on cards to be observed, in particular in order to make website categories which relate to the mental representation of site users.

Card sorting consists in presenting the user with a pack of "cards" (up to fifty the size of a playing card so they may be "played"), which may be of different kinds (formulated with headings, information categories, etc.); sorting may be carried out physically with a paper set or conceptually on computer, the cards being represented by words on the screen. Card sorting is carried out on a "user panel" of people who represent the target. The users may be seen in groups or individually. In groups (4 to 10 people), sorting has the advantage of being quicker to carry out and allowing more elaborate results to be obtained, since it takes advantage of the creative dynamic of the group by drawing each individual game to everyone's attention. On the other hand, individual expression with the card game means the influence of certain dominant members of the group is limited. Card ranking is a statistical activity based on the frequency with which concepts are associated with each other. It allows associations to be found but does not, however, give information of a hierarchical nature in relation to the concepts ranked.

In the field of care, Q methodology [19] combines qualitative and quantitative methods to identify attitudes, perceptions, feelings and values and to explore life experiences such as stress, self-esteem, body image, etc. [20]. In our study, only the frequency and order of appearance of the cards have already been studied, and there is no complete analysis of the contents of the discussions which followed.

Card sorting seemed to us appropriate for our context, as it allows:

- The expression of each participant to be facilitated regardless of his/her position within the group, even in the presence of a doctor or head of department.

- A selection of numerous pieces of information which are often similar and interlinked to be made and ranked quickly.

- Collegial discussion to be generated in reaction to the pieces of information put on the table, without judging the person bringing it up.

During team meetings, the "card sorting" method was quickly adopted by professionals as a tool to assist with discussion beyond the context of the study. This certainly implies that the discussions, despite not being structured, were very much of a collegial nature, which in theory leads to a decision with the agreement of participants in institutions admitting people suffering from very advanced dementia.

The participants were not, however, compelled to mention their feelings in relation to a case, and it is significant that the anonymity which we tried to maintain so that each person could feel "listened to" without value judgement was very often discarded by the individuals themselves. The card sorting method in groups was adopted after the study by several gerontology teams for their ordinary decisions [21].

## Competing interests

The authors declare that they have no competing interests.

## Authors' contributions

LP, CV, DFC, JLB, PP and RA developed the study concept. RA coordinated the study. LP and CV conducted the statistical analysis and developed the study design. SG and FS conducted the interviews. DFC, SG, FS, EC, JLB, PP and RA carried out the interpretation of the data. LP and RA supervised the interviews. LP and CV wrote the manuscript and all authors reviewed and approved it.

## Pre-publication history

The pre-publication history for this paper can be accessed here:

http://www.biomedcentral.com/1472-684X/10/4/prepub
